# Bubble CPAP Therapy in Newborns With Respiratory Distress With Its Outcome—A Prospective Observational Study From a Neonatal Intensive Care Unit of Tertiary Care Hospital in Western Nepal

**DOI:** 10.1155/ijpe/2200049

**Published:** 2025-10-14

**Authors:** Hanshmani Prasad Chaudhary, Binod Kumar Gupta, Dinesh Chaudhary, Raju Kafle, Nagendra Chaudhary

**Affiliations:** Department of Pediatrics, Universal College of Medical Sciences, Siddharthanagar, Lumbini, Nepal

**Keywords:** bubble CPAP, newborns, outcome, success and failure rates

## Abstract

**Background:**

Respiratory distress is one of the most common reasons for the admission of neonates to the NICU in low and middle-income countries (LMICs) like Nepal, where bubble CPAP therapy is one of the most emerging and first-line treatments.

**Aims:**

The aim is to study the various indications of bubble CPAP therapy in neonates with respiratory distress in different clinical conditions, success and failure rates, and outcomes.

**Methods:**

A prospective observational study was conducted for 17 months (Oct 2021–Feb 2023) at Universal College of Medical Sciences, Bhairahawa, Nepal, where 90 neonates with respiratory distress were enrolled and kept on bubble CPAP therapy. Various clinical conditions of CPAP use were observed along with success and failure rates. The final outcome was documented as discharge or death occurred.

**Results:**

A total of 90 babies with respiratory distress required CPAP therapy, where 53.3% were preterm, 44.4% term, and the rest postterm. About 32.2% of neonates had birth asphyxia, 16.7% had respiratory distress syndrome (RDS), 14.4% of cases had prematurity, and 12.2% had meconium aspiration syndrome. The study found a significant mean difference in Silverman score (in preterms) and Downe's score (in terms) before and after 6 h of CPAP (*p* < 0.001 in each) and 12 h of life (*p* < 0.001 in each). Out of the total enrolled babies, 67 (74.4%) neonates survived whereas 25.6% had mortality. The present study showed CPAP success and failure rates of 68.9% and 31.1%, respectively. Out of total CPAP failure rate (*n* = 28) babies, 67.9% had mortality and 32.1% were successfully discharged. No significant association of CPAP failure was seen when compared to gender, religion, gestational age, mode of delivery, resuscitation received, various indications of CPAP use, use of antenatal steroid, and duration of initiation of CPAP.

**Conclusion:**

Birth asphyxia, respiratory distress, and prematurity were three leading causes of respiratory distress for the initiation of CPAP in the present study.

## 1. Introduction

Respiratory distress (RD) is an important neonatal issue encountered in newborns soon after birth due to diverse underlying neonatal conditions originating from pulmonary and extrapulmonary disorders. It occurs in 15% of term and 29% of preterm babies requiring admission to the neonatal intensive care unit (NICU), leading to 20% of neonatal deaths [1]. The cardiorespiratory system must undergo a number of quick physiologic changes in order for the transition from fetal to newborn life to be successful [2]. Even if it may be temporary, prolonged distress necessitates a logical diagnosis and therapeutic approach to maximize results and minimize symptoms. The typical symptoms of RD include tachypnea, intercostal retraction, nasal flaring, grunting, and cyanosis.

Bubble CPAP, a noninvasive respiratory support method, is most commonly used to support the breathing of newborns that are experiencing RD. It is a noninvasive respiratory support method best suited in neonatal facilities in underdeveloped nations when resources are scarce for RD, which can be used with a conventional ventilator, a bubble circuit, a CPAP driver, a face mask, a nasopharyngeal tube, or nasal prongs [3]. b-CPAP works by maintaining a constant inspiratory and expiratory pressures above the ambient pressure and increases arterial oxygen content as it recruits collapsed alveoli to increase functional residual capacity, protects surfactant, and reduces right-to-left shunting by reducing ventilation–perfusion mismatch. It also decreases airway resistance by increasing pharyngeal cross-sectional area, thus lessening obstructive apnea, tachypnea, and the severity of central apnea. It decreases lung injury, work of breathing, and alveolar edema [4].

b-CPAP can be used in newborns with RD due to various reasons like perinatal asphyxia, RD syndrome, transient tachypnea of the infant, meconium aspiration syndrome, congenital pneumonia sepsis, and postextubation after mechanical ventilation. Additionally, it also provides respiratory support to newborns with tracheomalacia, bronchopulmonary dysplasia (BPD), and other airway abnormalities that are either congenital or acquired and likely to cause airway collapse.

The data on the use of b-CPAP therapy for various clinical scenarios in newborns with RD in Nepal is scarce. Also, there is a paucity of data on the success and failure rates of CPAP for those conditions along with its final outcomes. Therefore, the present study was conducted to find out different clinical conditions and success and failure rates along with its outcomes in newborns with RD requiring b-CPAP therapy, which can allow us in prompt interventions in such babies with RD.

## 2. Material and Methods

A prospective observational study was conducted in the NICU of Universal College of Medical Sciences (UCMS) for 17 months (Oct 2021–Feb 2023). Ethical clearance was obtained from the institute review committee (IRC) of UCMS. Sample size calculation was done considering the prevalence of the disease to be 4.2% at a 95% confidence interval with an allowable error of 5%.

Shreeyash acquatherm bubble CPAP (manufactured by Shreeyash Electro Medicals, India) was used to provide continuous positive airway pressure through a bubble mechanism that helps to maintain lung volume, improve oxygenation, and reduce the need for invasive ventilation. The device consists of an inbuilt compressor (for generating the required gas flow) that supports blended oxygen delivery with adjustable flow rates and FiO_2_ (fraction of inspired oxygen). The acquatherm technology of this device provides a water-based humidification and heating system to deliver warmed, humidified air–oxygen mixtures, preventing dryness and supporting patient comfort during therapy, making it suitable to be used in NICUs of resource-limited countries.

RD in term and preterm babies was assessed by Downe's score and Anderson–Silverman scoring system, respectively, where babies with scores of 3 or more received b-CPAP therapy. Ninety neonates with RD receiving b-CPAP therapy were enrolled in the study after obtaining written and verbal consents from the parents. Any death of a newborn with RD before being enrolled, any prior intubation and mechanical ventilation support required, newborns leaving against medical advice (LAMA) even after being enrolled, and parents of newborns not willing to give consent were excluded from the study. Baseline investigations were sent as per the hospital protocol and various clinical conditions of CPAP use were documented along with the final outcome (discharge and/or death). Data analysis was done using SPSS Software Version 16.0 (SPSS Inc., Chicago, Illinois). Descriptive statistics in terms of frequency, percentage, mean, and standard deviation were calculated. Analytical study was done using the chi-square test (for categorical variables) and the Student *t*-test (for numerical variables). A *p* value of ≤ 0.05 was considered statistically significant.

### 2.1. Definitions

RD is the presence of one or more of the following signs—tachypnea with a respiratory rate > 60 breaths/min, grunting, retractions, flaring ala-nasi, cyanosis, and/or apnea.

Success rate is defined as successful weaning from the b-CPAP and having spontaneous room air breathing.

Failure rate is the CPAP failure rate defined as worsening of RD and need for mechanical ventilation during the CPAP therapy. Any of the following events was considered as CPAP failure:

a. Requirement of pressure > 8 cm H_2_O
b. FiO_2_requirement > 0.6 (for 24 h or more)
c. PaO_2_ < 50 mm Hg on maximum acceptable settings
d. PaCO_2_ > 60 mm Hg and pH < 7.25 on maximum acceptable settings
e. Air leak on b-CPAP
f. Recurrent apnea on b-CPAP despite caffeine citrate and/or surfactant therapy (in preterm babies)

## 3. Results

Out of a total of 1145 admitted babies, 100 babies had RD at presentation to the NICU. Ninety babies with RD that were on CPAP therapy fulfilled the inclusion criteria and were included in the study. The total prevalence of RD in the present study was 8.7%. The enrolled babies had a male preponderance (72.2%) with a mean gestational age of 36 ± 3.3 weeks. The mean birth weight was 2250.8 ± 697 g, where 53.3% were preterm, 44.4% term, and the rest postterm. The baseline characteristics of enrolled babies along with their mothers are given in Table 1.

Prolonged rupture of membranes (PROM) was seen in 2.2% of mothers in the present study. Thyroid disorders (8.9%) and hypertension (7.8%) were predominant in the mothers of babies enrolled in the present study.

Out of a total of 90 enrolled patients with RD requiring CPAP therapy, 32.2% (*n* = 29) neonates had birth asphyxia, 16.7% (*n* = 15) had respiratory distress syndrome (RDS), and 14.4% of cases were premature as shown in Table 2. Other cases requiring CPAP therapy were meconium aspiration syndrome (12.2%), pneumonia (10%), sepsis (6.7%), and congenital heart disease (3.3%). Two cases each of pneumothorax and shock also required CPAP therapy in the present study.

The mean duration of CPAP therapy in the present study was 3.4 ± 2.4 days where 80% of the cases received CPAP within the first 6 h of life. More than half of the enrolled babies (55.6%) were weaned off from CPAP in 2–14 days, whereas 43.3% were weaned off within 6 h to 2 days. The study found a significant mean difference in Silverman score (in preterm babies) and Downe's score (in term babies) before and after 6 h of CPAP (*p* < 0.001 in each) and 12 h of life (*p* < 0.001 in each).

Out of total enrolled babies (*n* = 90), about two-thirds (*n* = 67, 74.4%) neonates survived and the rest had mortality. The present study showed CPAP success and failure rates of 68.9% and 31.1%, respectively.

The present study showed that about 82.6% of total deaths of babies were from the CPAP failure group and 67.8% of babies (*n* = 19) with CPAP failure received CPAP for 2–14 days.

Out of total CPAP failures (*n* = 28), 27 babies received invasive ventilatory support, whereas one could not be kept due to poor financial constraints and the unwillingness of the parents. This newborn was continued on b-CPAP only with increased setting parameters.

Out of the total CPAP failure group (*n* = 28) babies, 67.9% (*n* = 19) had mortality and 32.1% (*n* = 9) were successfully discharged. About 21.1% of babies had complications due to CPAP use, which are depicted in Table 3.

The present study did not find any significant association of CPAP failure when compared to gender, religion, gestational age, mode of delivery, resuscitation received, various indications of CPAP use, use of antenatal steroid, and duration of initiation of CPAP, as shown in Table 4.

## 4. Discussion

Worldwide, the majority of newborn babies are admitted to the NICU due to various clinical conditions leading to RD. The prevalence of RD in the present study was 8.7%, which may vary from 4.24% to 36.1% in various studies depending on the hospital setting and the country located [5, 6].

In the present study, the top three leading causes of RD requiring b-CPAP therapy were birth asphyxia, RDS, and prematurity, respectively. A study conducted by Haque et al. in Bangladesh showed that perinatal asphyxia was the third leading cause of RD, which was slightly less than in the present study (25% vs. 32.2%) [7]. Another study done by Raha et al. (Bangladesh) showed a further lower incidence of perinatal asphyxia (10%) leading to RD [8]. Although many studies conducted in different countries also suggest a lower incidence of birth asphyxia in comparison to the present study as the cause of RD, birth asphyxia still remains one of the top three leading indications for RD requiring respiratory support [9, 10]. On the other hand, studies conducted on preterm babies suggest that RDS is an important cause of CPAP therapy or invasive ventilation [11, 12].

A recent study conducted by Al-lawama et al. demonstrated transient tachypnea of the newborn (TTN) as the predominant cause (42%) of RD in newborns requiring CPAP therapy [13]. Another study conducted by Rijal and Shrestha from Nepal found that meconium aspiration syndrome (21.1%), septicemia (16.5%), TTN (15.5%), pneumonia (14.6%), and birth asphyxia (11.9%) were the important causes of RD in newborns [14]. The higher proportion of RD cases due to perinatal asphyxia in our setting could be due to females of LMICs not receiving adequate antenatal care and visits as recommended by the WHO in contrast to developed nations, which can worsen pregnancy and labor-related complications.

The mean duration of CPAP therapy in babies with RD in the present study was 3.4 ± 2.4 days, which was similar to a 6-month Nepalese study conducted by Manandhar [1]. b-CPAP-related complications in the present study were noticed in 21% of babies, the majority in the form of nasal injury, whereas Al-lawama et al. found a slightly lower proportion of b-CPAP-related complications (16%) in their study [13]. The results showed that there was significant improvement in Silverman score and Downe's score at 6 and 12 h after initiation of CPAP therapy in preterm and term babies with RD. A study conducted in Bangalore, India, by Parasuramappa and Belavadi also found similar results with significant decreases in DS when compared before and after 6 and 12 h of CPAP use in preterm infants with mild to moderate RDS [12].

The success rate of b-CPAP therapy for improving RD in this study was 69%, which was higher than the study conducted by Manandhar et al. (61%) [1]. Another study conducted in India demonstrated a higher success rate for weaning from CPAP [15]. This variability of success rate in various studies could be explained as the indications of CPAP use in their studies were disease-specific, whereas the present study analyzed the CPAP success or failure in babies with RD having multiple etiologies.

About 20%–40% of neonates initiated on CPAP might fail and require intubation and mechanical ventilation [16]. Murki et al. in their retrospective study found that 14.7% of babies had early CPAP failure and required mechanical ventilation within 72 h of birth [17]. The present study demonstrated that 30% of babies who received CPAP failed to improve and received invasive ventilation. Murki et al. also concluded that early starting of CPAP, InSurE, early surfactant administration, lower CPAP pressures, and lower FiO_2_ at the starting of CPAP were the important determinants of success. The present study did not find any significant association of CPAP failure or success when compared to gender, religion, gestational age, mode of delivery, resuscitation received, indications of CPAP use, use of antenatal steroid, and duration of initiation of CPAP. This necessitates further multicentric studies with a larger sample size to conclude the exact determinants of CPAP success and failure.

About two-thirds of the babies in the present study survived and were discharged. A study done by Kawaza et al. showed the overall survival rate of 71%, which was slightly lower than the present study [18]. Another study conducted in Malawi showed a lower survival rate (65%) than the present study [19]. On the other hand, a Kenyan study conducted by Myhre et al. had a higher survival rate (85%) in babies kept on CPAP [20]. Similarly, a study conducted in Jordan also showed a much better (93%) survival rate [13]. This variability in the survival rates could be because of various influencing factors like the NICU setup and facilities available, along with newborn parameters like gestational age, birth weight, and various etiological factors for RD.

Our study had few limitations. First, this was a hospital-based study done with a limited sample size. Therefore, the results may not reflect or represent the whole population. Hence, further multicentric studies with a larger sample size could give more accurate indications for CPAP use in babies presenting with RD in the NICU. Secondly, as we did not use any control group in the study, we are unable to discuss the comparative analysis of the efficacy of b-CPAP. We also did not compare the FiO_2_ and CPAP pressures between the CPAP success and failure groups.

## 5. Conclusion

Birth asphyxia, RDS, and prematurity were three leading causes of RD for the initiation of CPAP in the present study. There was a significant improvement in the Silverman score (in preterm babies) and Downe's score before and after the initiation of CPAP in babies with RD. The study did not find any correlation with various factors for CPAP failure, which necessitates the requirement of further multicentric studies with a larger sample size.

## Figures and Tables

**Table 1:** Baseline characteristics of the babies and their mothers enrolled (*n* = 90).

**Variables**	**N** **(%)**
Sex	
Male	65 (72.2)
Female	25 (27.8)
Religion	
Hindu	75 (83.3)
Muslim	15 (16.7)
Maturity	
Preterm (< 36 weeks and 6 days)	48 (53.3)
Term (37–42 weeks)	40 (44.4)
Postterm (42 weeks and more)	2 (2.2)
Mode of delivery	
NVD	47 (52.2)
LSCS	43 (47.8)
Type of pregnancy	
Singleton	79 (87.8)
Twin	11 (12.2)
Antenatal steroid received	
Yes	13 (14.4)
No	77 (85.6)
Antenatal steroid received (preterm)	
Yes	13 (27.1)
No	35 (72.9)

**Table 2:** Indications, time of starting, and duration of CPAP.

	**Frequency (** **n** **)**	**Percentage (%)**
Indications of CPAP use		
Birth asphyxia	29	32.2
Respiratory distress syndrome (RDS)	15	16.7
Prematurity	13	14.4
Meconium aspiration syndrome (MAS)	11	12.2
Pneumonia	9	10.0
Sepsis	6	6.7
Congenital heart disease (CHD)	3	3.3
Pneumothorax	2	2.2
Shock	2	2.2
Time of CPAP started		
< =6 h	72	80.0
> 6 h	18	20.0
Duration of CPAP needed		
6 h–2 days	39	43.3
2–14 days	50	55.6
15–28 days	1	1.1

**Table 3:** CPAP success and failure rates, complications due to its use, and outcomes of babies.

	**Frequency (** **n** **)**	**Percentage (%)**
CPAP failure		
Yes	28	31.1
No	62	68.9
Ventilator required		
Yes	27	30.0
No	63	70.0
Complications of CPAP		
Nasal septum injury	2	2.2
Nasal bridge injury	5	5.6
Eye puffiness and hyperemia	8	8.9
Abdominal distension	4	4.4
None	71	78.9
Outcomes of babies		
Discharged	67	74.4
Death	23	25.6

**Table 4:** Variables associated with CPAP failure.

**Variables**		**CPAP failure**		**p** **value**
	**Variables**	**Yes**	**No**	
Gender	Male	17 (26.2)	48 (73.8)	0.101
	Female	11 (44)	14 (56)	

Religion	Hindu	24 (32)	51 (68)	0.684
	Muslim	4 (26.7)	11 (73.3)	

Gestational age	Preterm	14 (29.2)	34 (70.8)	0.807
	Term	13 (32.5)	27 (67.5)	
	Postterm	1 (50)	1 (50)	

Modes of delivery	LSCS	15 (34.9)	28 (65.1)	0.46
	Vaginal delivery	13 (27.7)	34 (72.3)	

Pregnancy	Single	25 (31.6)	54 (68.4)	0.535
	Twins	3 (27.3)	8 (72.7)	

Indications of CPAP use	RDS	4 (26.7)	11 (73.3)	0.473
	MAS	4 (36.4)	7 (63.6)	
	Pneumonia	2 (22.2)	7 (77.8)	
	Birth asphyxia	10 (34.5)	19 (65.5)	
	Prematurity	4 (30.8)	9 (69.2)	
	Sepsis	1 (16.7)	5 (83.3)	
	Shock	2 (100)	0 (0)	
	CHD	1 (33.3)	2 (66.7)	
	Pneumothorax	0 (0)	2 (100)	

Resuscitation	Yes	8 (34.8)	15 (65.2)	0.659
	No	20 (29.9)	47 (70.1)	
	Yes	4 (30.8)	9 (69.2)	0.626
	No	24 (31.2)	53 (68.8)	

Antenatal steroids	< 6 HOL	23 (31.9)	49 (68.1)	0.733
	> 6 HOL	5 (27.8)	13 (72.2)	

CPAP started	6 h–2 days	9 (23.1)	30 (76.9)	0.217
	2–14 days	19 (38)	31 (62)	
	15–28 days	0 (0)	1 (100)	

## Data Availability

The data that support the findings of this study are available from the corresponding author upon reasonable request.

## References

[1] Manandhar S. R. (2019). Outcome of Respiratory Distress in Neonates With Bubble CPAP at Neonatal Intensive Care Unit of a Tertiary Hospital. *JNMA: Journal of the Nepal Medical Association*.

[2] Baseer K. A. A., Mohamed M., Abd-Elmawgood E. A. (2020). Risk Factors of Respiratory Diseases Among Neonates in Neonatal Intensive Care Unit of Qena University Hospital, Egypt. *Annals of Global Health*.

[3] Byram S. K., Sivaramakrishna Y., Raju M. (2019). Outcome of Bubble (CPAP) Continuous Positive Airway Pressure in Neonates with Respiratory Distress and Its Failure Factors. *International Journal of Contemporary Medical Research [IJCMR]*.

[4] Egesa W. I., Waibi W. M. (2020). Bubble Nasal Continuous Positive Airway Pressure (bNCPAP): An Effective Low-Cost Intervention for Resource-Constrained Settings. *International Journal Of Pediatrics*.

[5] Zaman S., Goheer L., Riaz H. (2013). Prevalence and Etiology of Respiratory Distress in Newborns. *Pakistan Armed Forces Medical Journal*.

[6] Bijow F. W. (2022). High Prevalence of Neonatal Respiratory Distress and Its Possible Etiologies in NICU in Syria. *International Journal of Pediatric Research*.

[7] Haque A., Baki M. A., Begum T., Akhter S., Begum S., Nahar N. (2013). Etiology of Respiratory Distress in Newborn Experience in BIRDEM. *BIRDEM Medical Journal*.

[8] Raha B. K., Alam M. J., Bhuiyan M. A. Q. (2021). Spectrum of Respiratory Distress in Newborn: A Study From a Tertiary Care Military Hospital. *Journal of Bangladesh College of Physicians and Surgeons*.

[9] Liu J., Yang N., Liu Y. (2014). High-Risk Factors of Respiratory Distress Syndrome in Term Neonates: A Retrospective Case-Control Study. *Balkan Medical Journal*.

[10] Aljawadi H. F. M., Ali E. A. A. M. (2019). Neonatal Respiratory Distress in Misan: Causes, Risk Factors, and Outcomes. *Iranian Journal of Neonatology*.

[11] Abdallah Y., Mkony M., Noorani M., Moshiro R., Bakari M., Manji K. (2023). CPAP Failure in the Management of Preterm Neonates with Respiratory Distress Syndrome Where Surfactant is Scarce. A Prospective Observational Study. A Prospective Observational Study. *BMC Pediatrics*.

[12] Parasuramappa H. S. C., Belavadi G. B. (2017). A Descriptive Study on the Use of Bubble CPAP in a Level 2 Neonatal Intensive Care Unit in Bangalore, India. *Sri Lanka Journal of Child Health*.

[13] Al-lawama M., Alkhatib H., Wakileh Z. (2019). Bubble CPAP Therapy for Neonatal Respiratory Distress in Level III Neonatal Unit in Amman, Jordan: A Prospective Observational Study. *International Journal of General Medicine*.

[14] Rijal P., Shrestha M. (2018). Analysis of Neonatal Respiratory Distress in Neonatal Intensive Care Unit at Nepal Medical College. *Journal of Nepal Health Research Council*.

[15] Urs P. S., Khan F., Maiya P. P. (2009). Bubble CPAP - a Primary Respiratory Support for Respiratory Distress Syndrome in Newborns. *Indian Pediatrics*.

[16] Thukral A., Sankar M. J., Chandrasekaran A., Agarwal R., Paul V. K. (2016). Efficacy and Safety of CPAP in Low- and Middle-Income Countries. *Journal of Perinatology*.

[17] Murki S., Kandraju H., Oleti T., Saikiran, Gaddam P. (2020). Predictors of CPAP Failure -10 Years’ Data of Multiple Trials From a Single Center: A Retrospective Observational Study. *Indian Journal of Pediatrics*.

[18] Kawaza K., Machen H. E., Brown J. (2014). Efficacy of a Low-Cost Bubble CPAP System in Treatment of Respiratory Distress in a Neonatal Ward in Malawi. *PLoS One*.

[19] Carns J., Kawaza K., Liaghati-Mobarhan S. (2019). Neonatal CPAP for Respiratory Distress Across Malawi and Mortality. *Pediatrics*.

[20] Myhre J., Immaculate M., Okeyo B. (2016). Effect of Treatment of Premature Infants With Respiratory Distress Using Low-Cost Bubble CPAP in a Rural African Hospital. *Journal of Tropical Pediatrics*.

